# Enhancing Nephrology Research with Natural Language Processing and Artificial Intelligence: A Case for More Comprehensive Symptom Documentation

**DOI:** 10.34067/KID.0000000755

**Published:** 2025-05-29

**Authors:** Rebecca J. Allen, Alferso C. Abrahams

**Affiliations:** 1 Center for IT Engagement (cITe), Mount St. Joseph University, Cincinnati, Ohio; 2 Department of Nephrology and Hypertension, University Medical Center Utrecht, Utrecht, The Netherlands

**Keywords:** dialysis, hemodialysis, patient self-assessment, patient-centered care, quality of life, artificial intelligence

Patients undergoing dialysis experience a significant symptom burden. Common symptoms include fatigue, muscle cramps, dry skin, itching, pain, restless legs, sleep disturbance, sexual dysfunction, nausea, diarrhea, difficulty concentrating, anxiety, and depression.^1^ This high symptom burden has a substantial negative effect on health-related quality of life.^2^ Thus, documentation and treatment of symptoms is imperative. The study in this issue by Dai *et al.*, “Natural Language Processing Identifies Under-Documentation of Symptoms in Patients on Hemodialysis,”^3^ highlights a concerning issue: symptom documentation is often inadequate. The study uses natural language processing (NLP), a subset of artificial intelligence (AI), to analyze electronic health records (EHRs). Because NLP and AI are not standard components of medical training, many health care professionals may be unfamiliar with their principles and applications. Given this gap, a brief overview of NLP is essential for understanding the study's findings and facilitating a broader discussion on the potential of NLP and AI in nephrology and medicine.

Gaining a deeper understanding of NLP and AI can provide valuable context for exploring the study by Dai *et al.* NLP is beginning to be more widely used in medicine, with one of its most common applications being the analysis of EHRs. More specifically, NLP approaches can be categorized into two main types: rule-based and deep learning–based (which falls under AI). The study by Dai *et al.* used a rule-based NLP approach, which relies on predefined grammatical rules and word lists. This method is known for its simplicity, transparency, and effectiveness, especially when working with smaller datasets. On the other hand, deep learning–based NLP takes a more complex approach, using large datasets to train algorithms that can automatically learn patterns. Although this offers greater accuracy and adaptability in handling intricate applications, it also requires significant computational resources and large amounts of data. In addition, deep learning models often function as “black boxes,” meaning their decision-making process is less transparent. Regardless of whether NLP is rule-based or deep learning–based, its primary advantage in nephrology research is NLP's ability to process and analyze large volumes of textual data at a scale that would be impractical for human reviewers. By leveraging NLP, researchers can efficiently identify patterns and insights that might otherwise remain hidden, making it a powerful tool in advancing medical knowledge and improving patient care.^4^

Returning to the study at hand, the Dai *et al.* research compared symptom documentation extracted from EHR progress notes against patient-reported surveys on the basis of the validated dialysis symptom index questionnaire, which served as the reference standard. As is common in NLP research, the authors used the Python programming language—specifically the spaCy library—to analyze their data. The NLP model demonstrated strong performance when validated against manual EHR review, achieving a sensitivity of 0.92, specificity of 0.95, positive predictive value of 0.75, and negative predictive value of 0.99. However, when assessed against patient surveys, sensitivity decreased to 0.58 (95% confidence interval [CI], 0.47 to 0.68) and specificity to 0.73 (95% CI, 0.48 to 0.89), while positive predictive value remained high at 0.92 (95% CI: 0.82 to 0.97) and negative predictive value declined substantially to 0.24 (95% CI, 0.14 to 0.38). In short, NLP was accurate at identifying symptoms when the symptoms were documented, but often symptom documentation in the EHRs was lacking.

One of the most compelling aspects of this study is its implicit challenge to the traditional reliance on EHRs as one of the primary data sources for NLP-based medical research. The study found that provider notes within EHRs frequently failed to capture symptoms that patients reported in surveys, raising important questions about the completeness and accuracy of EHR-derived data. Expanding beyond EHRs to incorporate additional data sources could offer a more comprehensive insight into dialysis patients' symptom burdens and highlight where the health care systems may be falling short in addressing their needs. Dai *et al.* used patient surveys as the ground truth, and indeed, these surveys may have been the most accurate representation of what patients were experiencing. Yet, patient-reported outcome measures (PROMs) can be tiresome or fatiguing, particularly for individuals with limited health literacy, and PROMs' effectiveness is dependent on how they are worded, their frequency, and their mode of delivery (paper or digital). More research is needed to refine these factors and ensure that PROMs are accessible, engaging, and effective for patients.^5^ In addition, integrating PROMs into clinical workflows presents logistical challenges—adding another step to patient visits may be difficult for already time-strapped clinicians.^5^ AI offers a promising improvement for optimizing both the development and implementation of PROMs. By leveraging AI, researchers may be able to refine the content, length, and frequency of PROMs to maximize response rates and improve symptom reporting. For example, AI-driven, time-sensitive, and risk-profile–specific electronic PROMs could be deployed, possibly through secure text messaging, allowing for a more targeted, just in time approach. Such an approach may allow for a tailored focus on symptoms that are most impactful on health-related quality of life and/or most likely to be underdocumented or undertreated, ultimately improving patient care while minimizing burden on both patients and providers.

The underdocumentation of symptoms in the study also challenges readers to think about common research practices surrounding EHRs. Often, EHRs are regarded as static systems that passively collect data, but their design and functionality deserve closer scrutiny. One systematic review estimates that physicians spend 37% of their workday interacting with EHRs, highlighting their central role in clinical practice.^6^ When it comes to symptom underdocumentation, Dai *et al.* point out that encounter time constraints, provider fatigue, and ineffective communication are significant contributing factors to underdocumentation in the EHRs with in the study. NLP and AI offer promising avenues for improvement. EHR templates could be streamlined and tested to determine which improvements are most helpful to clinicians. EHRs could also be modified to provide templates that support effective patient-centered communication models^7^—after all, what is not discussed cannot be documented. In addition, advances in NLP and AI have made it increasingly feasible to extract and summarize spoken data, presenting a transformative opportunity to reduce reliance on manual data entry in EHRs. Moving away from extensive typing requirements could fundamentally reshape clinical workflows, allowing providers to devote more time and attention to direct communication with patients and caregivers. Automated transcription and analysis could also ensure that what patients actually say is captured, as opposed to just the information busy providers are able to document—an improvement that might have profound implications for communication and addressing symptom underdocumentation. Despite these potential benefits and increasing technical feasibility, AI-powered and NLP-powered documentation approaches, while feasible and available, are largely underutilized.^8^ While questions about acceptability and regulatory frameworks may need further exploration, the high burden of clinician workload and persistent gaps in symptom documentation suggest that alternative approaches are worth considering.

This study paves the way for future research by demonstrating that NLP-based symptom extraction performs well compared with manual chart review. The study also highlights the utility of patient symptom surveys. From a technical standpoint, NLP and AI approaches are able to detect patterns and relationships that traditional statistical methods might miss due to time constraints or overlooked correlations. NLP and AI would also offer the ability to dynamically combine different data sources in real time—this ability to aggregate notes and surveys would likely provide a more complete picture of patient symptoms than any sole datasource. Furthermore, AI techniques can facilitate clustering analyses that uncover distinct patient subgroups, not only based on demographic variables, but also on overlapping factors such as symptom patterns, disease progression, health-related quality of life measures, and end-of-life care intensity.^9^ By revealing these nuanced relationships, NLP and AI could help improve symptom management strategies and inform more personalized approaches to nephrology care.

For many in the nephrology community, getting started with NLP and AI research remains a challenge, as these technologies are still relatively new to the field and rarely covered in medical education. Bridging this gap requires interdisciplinary collaboration—nephrologists can provide clinical expertise, whereas computer scientists can refine NLP algorithms to align with health care needs.^10^ However, although NLP and AI offer powerful tools for analyzing complex datasets, they are not inherently free of bias. The fairness and equity of NLP analyses depend on factors such as training data selection and research framing, which inevitably shape outcomes. Moving forward, dedicated interdisciplinary research initiatives will be essential to integrating NLP into nephrology effectively. As this study demonstrates, AI-driven tools can enhance symptom recognition and documentation in EHRs, perhaps ultimately improving patient care. Realizing this NLP's full potential demands careful implementation, methodological transparency, and ongoing efforts to mitigate biases in data-driven models.
